# I. Favorite Prescriptions of Distinguished Practitioners; II. A Treatise on Headache and Neuralgia; III. A Practical Treatise on Nervous Exhaustion

**Published:** 1889-03

**Authors:** 


I. **Favorite Prescriptions of Distinguished Practitioners, with Notes on Treatment.** Compiled from the published writings or unpublished records of Drs. Fordyce Barker, Roberts Bartholow, Samuel D. Gross, Austin Flint, Alonzo Clark, Alfred L. Loomis, F. J. Bumstead, T. G. Thomas, H. C. Wood, Wm. Goodell, J. M. Fothergill, N. S. Davis, J. Marion Sims, Wm. H. Byford, E. G. Janeway, J. M. Da Costa, Meredith Clymer, Brown-Sequard, M. A. Pallen, W. A. Hammond, etc. By B. W. PALMER, A. M., M. D. Pp. 256. New York: E. B. Treat, 771 Broadway. 1888.

II. **A Treatise on Headache and Neuralgia,** including Spinal Irritation and a Disquisition on Normal and Morbid Sleep. By J. LEONARD CORNING, M. A., M. D., Consultant in Nervous Diseases to St. Francis' Hospital; Fellow of the New York Academy of Medicine; Member of the New York Neurological Society; of the Society of Medical Jurisprudence and State Medicine; of the Medical Society of the State of New York, etc., etc. Author of "A Treatise on Hysteria and Epilepsy;" "Local Anesthesia;" "Brain Exhaustion, with some Preliminary Considerations on Cerebral Dynamics;" "Carotid Compression;" "Brain Rest, being a Disquisition on the Curative Properties of Prolonged Sleep," etc., etc. Illustrated; pp. 227. New York: E. B. Treat, 771 Broadway. 1888.

III. **A Practical Treatise on Nervous Exhaustion** (Neurasthenia). Its Symptoms, Nature, Sequences, and Treatment. By GEORGE M. BEARD, A. M., M. D., Fellow of the New York Academy of Medicine; of the New York Academy of Sciences; Member of the American Neurological Association, etc., etc. Edited, with notes and additions, by A. D. ROCKWELL, A. M., M. D., Professor of Electro-Therapeutics in the New York Post-Graduate Medical School and Hospital; Fellow of the New York Academy of Medicine; Member of the American Neurological Association, etc. 8vo; pp. 254. New York E. B. Treat, 771 Broadway. 1889.

I. These three books constitute the three latest volumes of the Medical Classics Series, and are uniform in size and style with the handsome volumes that have already preceded them.

Dr. Palmer's book, which is substantially a republication on an enlarged scale, is a compilation of the prescriptions of the various authors named above. In some instances, it may be well to preserve this kind of literature in book form; but we have very little to say in commendation of "favorite prescriptions," no matter from what sources they may be obtained. We are, however, free to confess that of all the books of this sort we have seen, Dr. Palmer's is the most attractive. There are blank inter-leaves for the addition of such prescriptions as the reader may find useful.

II. Dr. Corning's treatise on Headache and Neuralgia is certainly a readable book, and it is doubtful if any person can arise from its careful perusal without the consciousness of having acquired some new information on these subjects. The general division of headache into intra- and extra-cranial, is a very satisfactory way to begin the study of the subject; and the sub-divisions which he makes of these two general heads are such as to lead one into paths of satisfactory knowledge. Dr. Corning's classification of neuralgia follows the well-established precedents, and is, perhaps, as good as any. He deals succinctly with the varieties, causes, diagnosis, pathology, prognosis, and treatment of this subtle and sometimes obstreperous disease, emphasizing local medication and electricity in its management. Parts IV. and V., on Irritative Conditions of the Spine, and Normal and Morbid Sleep, are well worth the price of the book to anyone whose library is deficient in such literature.

III. The re-issuing of this book in the present form is wise, in that it brings it nearer to the mass of physicians who cannot fail to profit by its careful study; and, again, in affording the editor an opportunity to make such needed additions as seemed necessary to bring it up to the level of the present state of medical thought.

Now-a-days it has become fashionable for the people to be so generally afflicted with "nervous prostration," that it is important for every physician to carefully study the various phenomena of its manifestations, and the countless guises under which it masquerades. The true and the false must be most cautiously differentiated, else errors of great magnitude will be resultant in treatment, and delays in cure consequently ensue.

In dealing with the diagnosis of neurasthenia, this book is particularly strong, and the editor has fittingly supplemented the work of the author in his own contributions thereto.

The treatment of nervous exhaustion is fully and elaborately considered, not only in relation to the administration of drugs, but all the accessories thereto, such as electricity, baths, massage, traveling, hygienic care, etc., are dwelt upon in detail. Throughout the text, cases are briefly referred to which forcibly illustrate the teachings of the author. It is a good book to possess.

